# One-Dimensional Position Detection Using a Cable Piezoelectric Sensor

**DOI:** 10.3390/s26134303

**Published:** 2026-07-07

**Authors:** Yusuke Yamazoe, Kento Ise, Sayaka Kohno, Junei Kobayashi, Takashi Nakajima

**Affiliations:** 1 Department of Applied Physics, Tokyo University of Science, 6-3-1 Niijuku, Katsushika-ku, Tokyo 125-8585, Japanjunei@rs.tus.ac.jp (J.K.); 2 Fujikura Ltd., 1-5-1 Kiba, Koto-ku, Tokyo 135-8512, Japan

**Keywords:** cable sensor, flexible piezoelectric sensor, one-dimensional sensing, position detection, polyvinylidene fluoride (PVDF)

## Abstract

This study established a sensor structure and readout circuit for identifying the point of force application within a single cable piezoelectric sensor. The sensor is highly flexible and enables wide-area sensing because it can be fabricated in long, continuous form. To determine the point of force application, the central electrode of the cable sensor was designed to have high resistance, and charge amplifiers were connected to both ends of the cable. The generated charge was divided and measured by the two amplifiers in a proportion corresponding to the point of force application. The position was predicted using a normalized ratio calculated from the charge quantities measured by the two amplifiers. The principle was first verified by accurately identifying the loaded section in a five-segment cable connected through discrete resistors. For cable sensors with a high-resistance central electrode, we predicted the point of force application with a root mean square error of 25 mm by interpolating the relationship between the point of force application and the normalized charge ratio with a smoothing-spline calibration model.

## 1. Introduction

Recent advances in the Internet of Things (IoT) have increased the demand for flexible and large-area sensing technologies [1,2]. Among the various sensing approaches proposed, organic piezoelectric sensors have attracted considerable attention because of their flexibility, biocompatibility, and fast response [3,4,5,6]. These characteristics have enabled a wide range of applications, including wearable devices [7,8,9,10], tactile sensors [11,12,13,14], and structural health monitoring [15,16,17,18].

Representative structures of organic piezoelectric sensors include island-type sensors, which detect mechanical loading at a single point, and matrix-type sensors, which acquire spatial distributions over a surface. While island-type sensors are suitable for localized, high-sensitivity measurements, large-area sensing requires deploying many individual elements, leading to increased wiring complexity and installation costs [19,20]. Matrix-type sensors share part of their electrodes, which helps suppress the increase in wiring; however, higher spatial resolution inevitably requires more wiring, and installation and implementation costs remain significant challenges for large-area applications [21,22]. Therefore, a new sensing architecture capable of covering wide areas without increasing the number of elements or wiring is highly desirable.

Against this background, distributed sensing technologies capable of covering wide areas with a single continuous sensing element have attracted considerable attention. In particular, distributed optical fiber sensing has been widely applied to structural health monitoring for large-scale infrastructure such as bridges, tunnels, pipelines, and transportation systems [23,24,25,26,27]. These approaches enable continuous strain or vibration monitoring with high spatial resolution over long distances. However, optical-fiber-based sensing systems generally require sophisticated optical interrogation equipment, precise installation conditions, and complex signal-processing procedures, thereby increasing implementation costs and system complexity [28].

In contrast, piezoelectric materials exhibit excellent stress sensitivity and rapid dynamic response because electrical charge is directly generated by mechanical deformation. Owing to these characteristics, piezoelectric materials have been widely utilized in applications such as touch panels, microphones, accelerometers, and sonar systems. In particular, piezoelectric sensing is highly advantageous for detecting subtle and localized mechanical stimuli.

Cable piezoelectric sensors, which can be fabricated in long and flexible forms, therefore represent a promising platform for high-sensitivity wide-area sensing [29,30,31]. Nevertheless, localization within a single piezoelectric cable sensor remains challenging. In conventional piezoelectric cable sensors, the entire sensing element behaves electrically as a distributed capacitive structure, and the generated charge is typically collected as an integrated signal over the entire cable. Therefore, local mechanical deformation cannot be directly mapped to spatial information by conventional readout methods. Consequently, methods capable of continuously identifying the local position of force application within a single continuous piezoelectric cable have not yet been fully established.

Existing approaches based on vibration propagation or voltage attenuation provide only limited localization capability [32,33]. Therefore, a new electrical localization principle that enables continuous position detection using only the electrical response of a single cable piezoelectric sensor is highly desirable.

In this study, a new localization method is proposed to continuously estimate the point of force application within a single cable piezoelectric sensor by utilizing charge division in a high-resistance central electrode. By intentionally increasing the electrode resistance and simultaneously measuring the generated charge at both ends of the cable with two charge amplifiers, the position of mechanical loading can be determined from the normalized charge ratio. Unlike conventional propagation-based methods, the proposed approach directly utilizes the quasi-static electrical charge distribution generated inside the sensor itself, enabling continuous one-dimensional localization without additional sensing elements, pulse-propagation analysis, or complex optical systems. This study presents the theoretical model, experimental validation, and calibration method of the proposed sensing architecture.

## 2. Materials and Methods

### 2.1. Cable Piezoelectric Sensor and Charge Measurement Circuit

**Figure 1:**
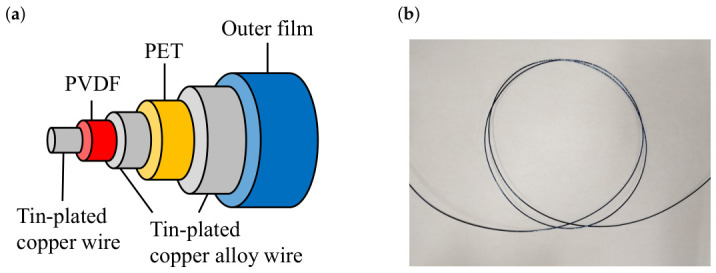
Internal structure and appearance of the cable piezoelectric sensor. (**a**) Internal coaxial structure. (**b**) Appearance of the fabricated cable sensor.

The cable piezoelectric sensor used in this study was manufactured by Fujikura Ltd. (Tokyo, Japan). Its internal structure is coaxial, as illustrated in Figure 1a. A piezoelectric polyvinylidene fluoride (PVDF) core is surrounded by polyethylene terephthalate (PET) as a dielectric layer and tin-plated copper-alloy wire. This coaxial configuration suppresses the influence of external electromagnetic noise and enables the stable acquisition of the charge generated by mechanical deformation. A photograph of the actual sensor appearance is shown in Figure 1b. The sensor has a diameter of 0.5mm and a mass density of 0.8g/m. Owing to its flexibility and uniform geometry, the sensor can be embedded or attached onto various surfaces without significantly altering the mechanical boundary conditions. 

**Figure 2:**
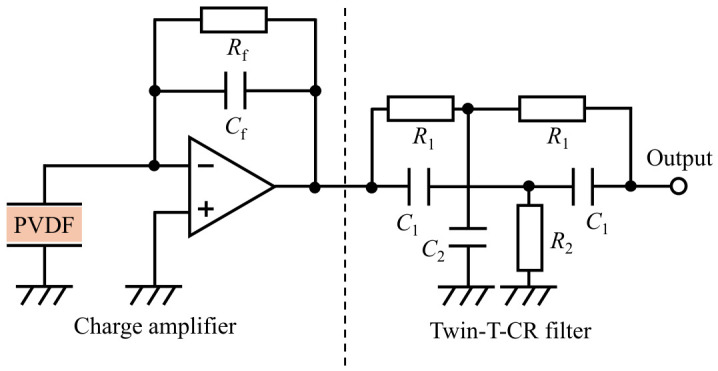
Schematic of the circuitry used for measuring the output of the cable piezoelectric sensor.

Figure 2 shows the measurement circuitry used in this study, which consists of a charge amplifier (CA) followed by a Twin-T-CR filter. The charge amplifier is essential to reading the charge accumulated in the piezoelectric element. It operates by accumulating an amount of charge equal to that generated by the piezoelectric element within a feedback capacitor ($C_{f}$) to maintain the virtual short at the operational-amplifier inputs. In this study, $C_{f}=100\;\mathrm{pF}$ and $R_{f}=1\;G\Omega$ were adopted as the feedback components. The Twin-T-CR filter functions as a 50Hz notch filter to suppress noise originating from the power supply, which is critical to high-sensitivity charge measurement. In this study, $C_{1}=100\;\mathrm{nF}$, $C_{2}=220\;\mathrm{nF}$, $R_{1}=31.6\;k\Omega$, and $R_{2}=15.82\;k\Omega$ were adopted. This circuit was connected to both ends of the cable, and the charge generated on the PVDF element was read as the sum of the charges measured by the two charge amplifiers. 

The charge amplifier circuits were constructed using LF356 operational amplifiers, powered by a dual ±15V supply. The generated signals were digitized using a National Instruments USB-6210 analog-to-digital converter with a 16-bit resolution, an input range of ±10V, and a maximum sampling rate of 250kS/s.

No additional high-pass or low-pass analog filters were applied in the measurement system. Instead, a Twin-T-CR notch filter was inserted after the charge amplifier stage to suppress 50Hz commercial power-line noise. Digital smoothing or post-processing filters were not applied unless otherwise stated.

The generated electrical charge was calculated from the output voltage of the charge amplifier according to

(1) $$Q=-C_{f}V_{\mathrm{out}}-\int \frac{V_{\mathrm{out}}}{R_{f}}\;dt,$$

where $C_{f}$ and $R_{f}$ are the feedback capacitance and feedback resistance, respectively. The first term corresponds to the charge stored in the feedback capacitor, while the second term represents the integrated leakage current flowing through the feedback resistor. The feedback resistor was introduced to prevent charge accumulation caused by the operational amplifier’s bias current and to stabilize long-term operation of the charge amplifier. The calculated charge was expressed in units of coulombs.

The cable sensor employed a PVDF piezoelectric film as the active sensing material. Typical material parameters reported for stretched and poled PVDF films include piezoelectric coefficients of $d_{31}\approx d_{32}=20$–30pC/N and $d_{33}=-20$ to −35pC/N, relative permittivity $\varepsilon_{r}\approx 10$, and Young’s modulus $1/s_{11}\approx 3\;\mathrm{GPa}$. These values are consistent with previously reported electromechanical properties of piezoelectric PVDF materials [34,35].

### 2.2. Charge-Division Model for Position Detection

For the PVDF layer in the cable sensor, the direct piezoelectric effect can be expressed using the linear constitutive relation

(2) $$D_{3}=d_{31}T_{1}+d_{32}T_{2}+d_{33}T_{3}+\varepsilon_{33}E_{3},$$

where $D_{3}$ is the electric displacement in the polarization direction; $T_{1}$, $T_{2}$, and $T_{3}$ are the stress components acting on the PVDF layer; $d_{31}$, $d_{32}$, and $d_{33}$ are the corresponding piezoelectric coefficients; $\varepsilon_{33}$ is the dielectric permittivity; and $E_{3}$ is the electric field. Because the PVDF film is wound around the central electrode to form the cable structure, the local stress state under external loading is not purely uniaxial, and both in-plane and thickness-direction stress components may contribute to the generated charge.

In this study, the detailed stress distribution in the wound PVDF layer was not explicitly solved. Instead, the generated charge was treated as the total piezoelectric charge produced by the local stress distribution near the loading point. The proposed position-detection model then describes how this generated charge is divided between the two charge amplifiers through the resistance distribution of the high-resistance electrode.

In the actual cable structure, the mechanical stress distribution inside the wound PVDF layer is influenced not only by the external loading force but also by cable attachment conditions, fixture stiffness, bending deformation, and local contact geometry. Therefore, the stress state in the cable is generally nonuniform and multidirectional.

In this study, these attachment-induced stress distributions were not explicitly modeled. Instead, the generated piezoelectric response was treated as an effective localized charge source produced near the point of force application. The proposed analytical model then focuses on the subsequent charge-division behavior driven by resistance in the high-resistance electrode. Despite this simplification, the experimental results demonstrated a stable monotonic relationship between the normalized charge ratio and the loading position.

**Figure 3:**
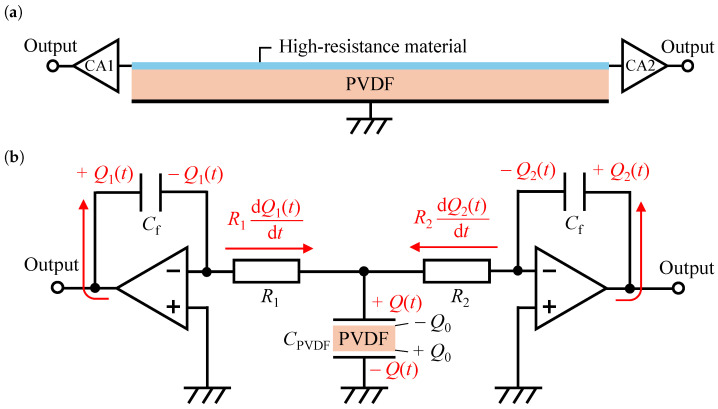
Proposed position detection architecture and equivalent circuit. (**a**) Sensing architecture using charge amplifiers connected to both ends of the cable. (**b**) Equivalent circuit model of the charge-division principle.

One-dimensional position detection within a single cable piezoelectric sensor is realized by employing a high-resistance central electrode and simultaneously acquiring electrical signals from both ends of the cable by using two charge amplifiers (CA1 and CA2). The overall sensing architecture and its equivalent circuit model are shown in Figure 3a,b, respectively. When a force is applied to the cable at position *x*, the generated charge is distributed to CA1 and CA2 according to the resistance ratio between the point of force application and each end. The two charge amplifiers are connected to the central electrode through resistances $R_{1}$ and $R_{2}$, which correspond to the electrode lengths from the point of force application to each CA. The resistance values are proportional to the corresponding lengths:

(3) $$R_{1}\propto x,\;R_{2}\propto \left(L-x\right),$$

where *L* is the total length of the cable and *x* is the distance from CA1.

When a force is applied to the cable, the PVDF element generates surface charge $Q_{0}$. In the standard operation of a charge amplifier, the output terminal supplies an equal and opposite amount of charge to the feedback capacitor ($C_{f}$) to compensate for the charge generated by the piezoelectric element and maintain the virtual short at the operational-amplifier inputs. In this study, this compensating charge is collectively supplied by CA1 and CA2.

Let Q(t) denote the total charge accumulated on the sensor electrodes, which ultimately compensates for the charge generated on the PVDF element. The total compensating charge, Q(t), is the sum of the charges ($Q_{1}\left(t\right)$ and $Q_{2}\left(t\right)$) supplied by the two charge amplifiers, CA1 and CA2, respectively:

(4) $$Q\left(t\right)=Q_{1}\left(t\right)+Q_{2}\left(t\right).$$

During charge compensation by the charge amplifiers, these charges flow through $R_{1}$ and $R_{2}$, respectively, causing voltage drops across the resistors. Considering the virtual short-circuit of the operational amplifier, the voltage-balance law dictates that this voltage drop is equal to the voltage drop generated by the sensor. Therefore,

(5) $$\begin{array}{cc} \frac{Q_{0}-Q\left(t\right)}{C_{\mathrm{PVDF}}} & =R_{1}\frac{dQ_{1}\left(t\right)}{dt}, \end{array}$$

(6) $$\begin{array}{cc} \frac{Q_{0}-Q\left(t\right)}{C_{\mathrm{PVDF}}} & =R_{2}\frac{dQ_{2}\left(t\right)}{dt}, \end{array}$$

where $C_{\mathrm{PVDF}}$ is the capacitance of the cable piezoelectric sensor.

Solving the equation set comprising (4), (5), and (6) provides the theoretical prediction for the charge accumulated in each CA feedback capacitor:

(7) $$\begin{array}{cc} Q_{1}\left(t\right) & =\frac{R_{2}}{R_{1}+R_{2}}Q_{0}\left\{1-\exp\left(-\frac{t}{C_{\mathrm{PVDF}}R_{\|}}\right)\right\}, \end{array}$$

(8) $$\begin{array}{cc} Q_{2}\left(t\right) & =\frac{R_{1}}{R_{1}+R_{2}}Q_{0}\left\{1-\exp\left(-\frac{t}{C_{\mathrm{PVDF}}R_{\|}}\right)\right\}, \end{array}$$

where $R_{\|}$ is the equivalent resistance defined by $1/R_{\|}=1/R_{1}+1/R_{2}$.

From (7), the measured charges $Q_{1}\left(t\right)$ and $Q_{2}\left(t\right)$ are in inverse proportion to resistances $R_{1}$ and $R_{2}$:

(9) $$\frac{Q_{2}\left(t\right)}{Q_{1}\left(t\right)}=\frac{R_{1}}{R_{2}}.$$

Since $R_{1}$ and $R_{2}$ are directly proportional to distances *x* and L−x, respectively, the ratio of the measured charges directly provides the ratio of the distances:

(10) $$\frac{Q_{2}\left(t\right)}{Q_{1}\left(t\right)}=\frac{x}{L-x}.$$

To establish a metric linearly correlated with position *x*, the normalized charge ratio, α, is defined as

(11) $$\alpha =\frac{Q_{1}\left(t\right)}{Q_{1}\left(t\right)+Q_{2}\left(t\right)}.$$

Substituting the distance relationship into this ratio yields

(12) $$\alpha =\frac{L-x}{L}.$$

Therefore, by calculating the normalized charge ratio from the two CA outputs, the point of force application, *x*, can be continuously and linearly determined along the cable:

(13) x=L(1−α).

This principle enables the continuous estimation of the point of force application without the need for additional sensors or complex pulse-based methods, confirming the feasibility of the proposed architecture.

### 2.3. Experimental Setup and Loading Conditions

**Figure 4:**
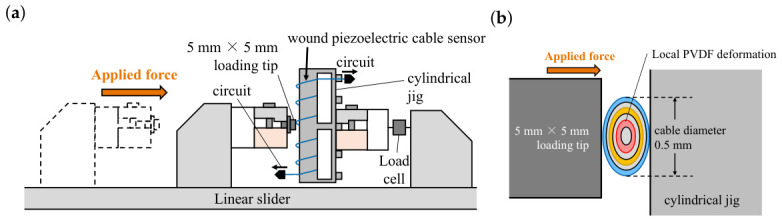
Experimental configuration of the cable piezoelectric sensor. (**a**) Overall experimental setup. (**b**) Enlarged schematic of the loading region showing the local deformation generated by the applied compressive force.

Figure 4 illustrates the experimental configuration of the cable piezoelectric sensor. The cable sensor was wound around a cylindrical jig and fixed during the measurements to maintain a consistent attachment condition. A compressive force was applied vertically to arbitrary positions along the cable using a linear-slider loading system equipped with a 5mm×5mm flat loading tip.

An enlarged schematic of the loading region is also shown to clarify the local contact geometry between the loading tip and the cable sensor. The figure illustrates how the applied compressive force locally deforms the wound cable structure and generates piezoelectric charge in the PVDF layer.

The loading experiments were conducted using a linear-slider loading system equipped with a flat loading tip. The loading tip had a square contact surface of 5mm×5mm. Because the cable sensor had a cylindrical geometry with a diameter of 0.5mm, the actual contact width along the cable varied depending on the local contact geometry and was estimated to range from approximately 5mm to 7mm, corresponding to the side length and diagonal length of the loading tip, respectively. Therefore, the effective contact area between the loading tip and the cable sensor was estimated to be approximately 2.5–$3.5\;{\mathrm{mm}}^{2}$.

A compressive load of 20N was applied and maintained for approximately 4s at each loading position using a load cell (TCSS-5L, Toyo Sokki Co., Ltd., Yokohama, Japan, rated capacity: 1–50 N). The force was applied as a quasi-static constant load rather than as an impulse load, resulting in a plateau region in the measured charge waveforms.

The loading experiment was repeated 20 times for each section to evaluate the reproducibility of the charge distribution response. The sampling rate for data acquisition was set to 50Hz.

### 2.4. Five-Segment Validation Cable

**Figure 5:**
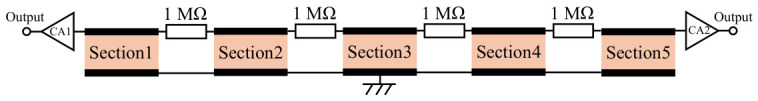
Experimental configuration for validating the charge-division principle using five cable segments connected in series with discrete resistors.

To validate the charge-division principle under a controlled resistance distribution, a single cable was divided into five sections and connected in series through discrete resistors, as shown in Figure 5. A force was applied to each section individually, and the measured charge distribution was used to identify the loaded section.

### 2.5. High-Resistance Electrode Cable and Calibration

To continuously predict the point of force application along the cable, a specialized sensor featuring a high-resistance central electrode was adopted. In this study, Tyranno fiber was employed as the central electrode material. This fiber is primarily composed of silicon carbide (SiC), and its electrical resistivity is controlled by adjusting the content of titanium, zirconium, carbon, and oxygen. The resistivity (ρ) of the specific fiber used in this experiment is approximately $10^{6}$Ω·m, which is several orders of magnitude higher than that of conventional metal electrodes. This design is essential because the position estimation principle relies on the assumption that electrode resistance is a continuous function of length, which allows the charge ratio to linearly map to the point of force application, as described by (3).

The high-resistance electrode cable used in the experiment was 1m long. Three individual 1m cables were used to assess reproducibility and material uniformity. Using this sensor, the point of force application was predicted along the 1m length according to the charge-division principle described in Section 2.2.

## 3. Results

### 3.1. Validation of the Charge-Division Model Using a Five-Segment Cable

**Figure 6:**
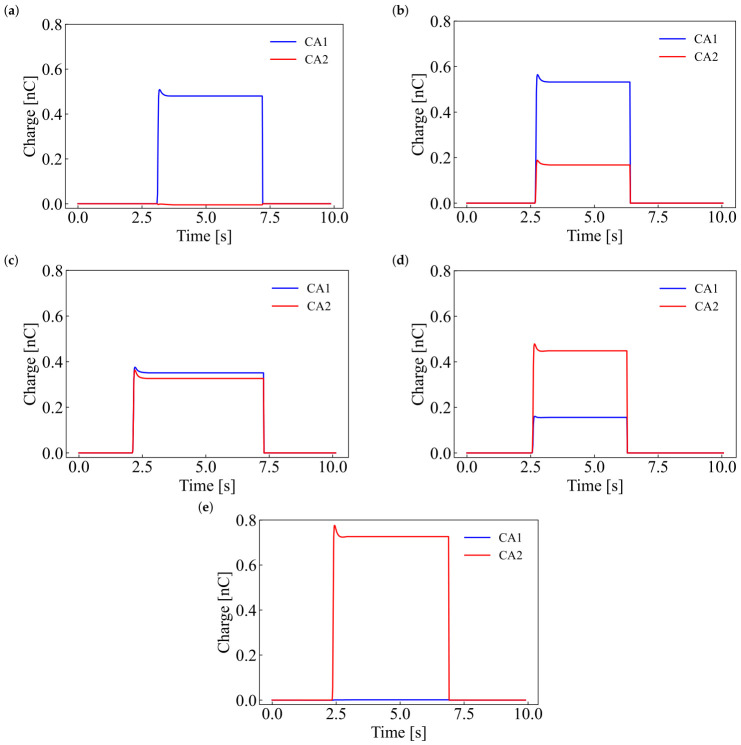
Measured charge waveforms for the five loaded sections. (**a**) Section 1. (**b**) Section 2. (**c**) Section 3. (**d**) Section 4. (**e**) Section 5.

To validate the principle of position detection, the experimental setup shown in Figure 5 featuring a single cable divided into five sections was constructed. A force was then applied to each section individually. The resulting charge waveforms are shown in Figure 6. In all measurements, the charge-response waveforms exhibit a plateau region during the loading period, indicating that the applied force was maintained approximately constant for several seconds. By contrast, transient behavior was observed during the rising edge immediately after contact formation, which is attributed to inertial effects and mechanical settling of the linear slider mechanism. It can be observed that the distribution of charges read by CA1 and CA2 varies significantly depending on the loaded section. In Sections 1 and 2, which are closer to CA1, the charge amount read by CA1 is greater than that read by CA2. Conversely, in Sections 4 and 5, the charge amount read by CA2 exceeds that read by CA1. In Section 3, the charges read by both channels are approximately equal, consistent with the fact that the resistance values from the point of force application to each charge amplifier are identical.

Based on these results, the normalized charge ratios were calculated. In (7), resistances $R_{1}$ and $R_{2}$ are represented as integer multiples of the discrete resistors placed between sections, enabling the determination of the ideal normalized ratios. The experimental ratios were derived from the waveforms in Figure 6, and the section whose ideal ratio was the closest to the measured value was identified as the predicted loaded section. The measured normalized ratios were extracted from the plateau region of the charge waveforms shown in Figure 6. Specifically, the charge values measured by CA1 and CA2 were averaged over the time window in which the applied load was maintained approximately constant. The normalized ratio was then calculated using (11) as $Q_{1}/\left(Q_{1}+Q_{2}\right)$ from the averaged plateau charges. Therefore, the ratios listed in Table 1 are not peak values or integrated values over the entire waveform but represent the charge distribution under the quasi-static loading plateau. These results are summarized in Table 1, which shows that the loaded sections were correctly predicted in all cases. Furthermore, the difference between the ideal and measured values remained within 0.02 for every section. Since this deviation is sufficiently small compared with the 0.25 interval between the ideal ratios of adjacent sections, the experimental data faithfully reproduce the theoretical predictions. These results experimentally demonstrate that the proposed model, where the generated charge is distributed in inverse proportion to the resistance from the point of force application to the two charge amplifiers, is the dominant principle in the actual sensing system.

### 3.2. Continuous Position Detection Using a High-Resistance Electrode Cable

**Figure 7:**
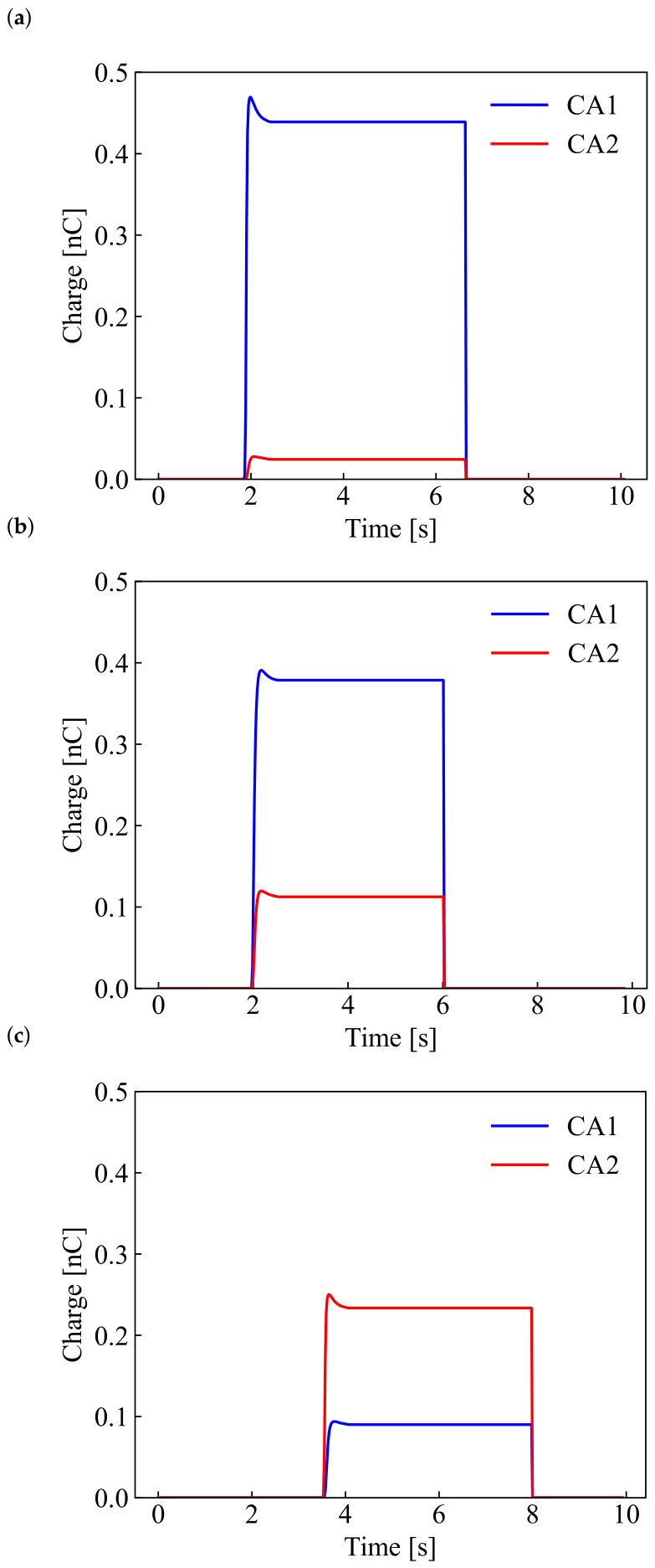
Charge waveforms at three points of force application along the high-resistance cable: (**a**) 105mm from CA1; (**b**) 520mm from CA1; (**c**) 905mm from CA1.

To evaluate the continuous position detection performance, experiments were conducted using a 1m cable sensor with a high-resistance Tyranno fiber electrode. Figure 7 shows representative charge waveforms obtained at three different points of force application—105mm in the top panel, 520mm in the middle panel, and 905mm in the bottom panel—all measured with CA1.

Ideally, at the midpoint of the cable (500mm), the charges read by CA1 and CA2 should be approximately equal. However, as shown in the middle panel of Figure 7, the charge amount at 520mm was significantly higher in CA1 than in CA2. Furthermore, even at the 905mm position shown in the bottom panel of Figure 7, which is extremely close to CA2, the charge amount in CA1 remained approximately half of that in CA2. These results indicate that the resistivity of the Tyranno fiber used in this study is nonuniform along the electrode length; thus, the resistance and the distance from the terminal do not show a simple linear relationship.

**Figure 8:**
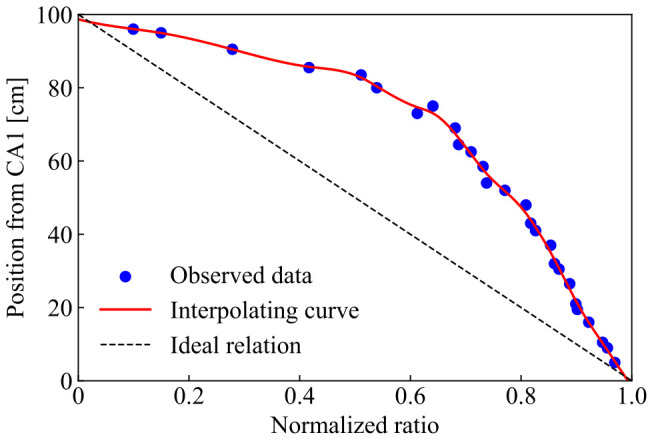
The normalized charge ratio as a function of the point of force application.

Figure 8 presents the relationship between the normalized charge ratio and the point of force application derived from 27 measurement points. The observed data shown in Figure 8 correspond only to the training dataset used for smoothing-spline fitting in each cross-validation cycle, while the excluded subset was used exclusively as the test dataset for prediction evaluation. The dashed line represents the ideal linear relationship expected when the electrode resistance is perfectly proportional to its length. However, the experimental data points significantly deviate from this ideal line, particularly showing a nonlinear shift where the equal-charge point moved toward the 700mm mark. This discrepancy is primarily attributed to the nonuniform resistivity distribution of the Tyranno fiber electrode, which arises from subtle variations in its chemical composition along the cable. This inherent material inconsistency necessitates a data-driven calibration approach rather than a simple linear approximation. To establish an accurate and robust calibration model, the smoothing-spline method was employed. The solid red line in Figure 8 represents the resulting interpolating curve, which functions as the characteristic calibration mapping for the sensor.

To evaluate the prediction accuracy, nine of the 36 measurement points were excluded from the spline fitting and used as test data in each validation cycle. The points of force application were estimated by inputting the measured normalized ratios into the derived spline function. To ensure the generalizability of the model, fourfold cross-validation was performed on the total dataset of 36 points, yielding a root mean square error of 2.5cm.

For the nine test data points, additional statistical analysis was conducted using the absolute prediction errors between the actual loading positions and the positions estimated from the smoothing-spline calibration curve derived from the 27 training points. The mean absolute error, maximum error, and standard deviation were 1.9cm, 8.3cm, and 1.6cm, respectively. The corresponding 95% confidence interval ranged from 0.6cm to 3.1cm.

For the fourfold cross-validation, the total dataset consisting of 36 measurement points was divided into four subsets using an interleaved assignment method. Specifically, the measurement points were sequentially allocated to the four subsets in a cyclic manner from the cable end. Therefore, the folds were not randomly generated but were systematically distributed over the entire cable length to reduce strong spatial bias in the calibration dataset.

This division method ensured that each fold contained measurement points distributed across the cable length, including data from all cable sections used in the experiments. During each validation step, one subset was used as the test dataset, while the remaining three subsets were used for smoothing-spline calibration. The test points were excluded from the spline fitting procedure during each validation cycle.

The purpose of this cross-validation was to evaluate the interpolation and generalization performance of the proposed localization method within the measured cable region rather than strict extrapolation performance outside the calibrated range.

Given the 1m total length of the cable, this corresponds to a relative error of only 2.5%. These results demonstrate that the proposed sensing architecture, combined with spline-based calibration, successfully enables high-precision localization even when high-resistance electrodes with nonuniform properties are used.

## 4. Discussion

The five-segment validation experiment confirmed the basic charge-division principle independently of the nonuniformity introduced by a continuous high-resistance electrode. The measured normalized ratios were separated by a margin that was much larger than the observed deviation from the theoretical values, which supports the use of the charge ratio as a position-dependent signal feature.

The experiment on the continuous high-resistance cable also showed a limitation that is important for practical implementation. The relationship between position and normalized charge ratio was not linear, indicating that the actual electrode resistance was not uniformly distributed along the cable. In the ideal case described by (3), the electrode resistance is assumed to be uniformly proportional to the cable length, and equal charge division ($Q_{1}=Q_{2}$) is expected when the point of force application is located at the midpoint of the cable (500mm).

However, the experimental results showed that the equal-charge condition appeared near 700mm, indicating that the actual resistance distribution along the Tyranno fiber electrode was asymmetric. This result suggests that the resistance per unit length was not constant along the cable. As a consequence, the condition $R_{1}=R_{2}$ was satisfied at a position shifted from the geometrical center. Therefore, the charge division was governed not simply by physical distance but by the accumulated electrical resistance between the loading point and each charge amplifier.

Quantitatively, the ideal model predicts the equal-charge point at 500mm, whereas the experimentally observed value shifted by approximately 200mm toward the CA2 side. This deviation demonstrates the influence of longitudinal resistance nonuniformity on the charge-distribution behavior and highlights the necessity of calibration for practical implementation.

Nevertheless, despite the nonlinear and asymmetric resistance distribution, the normalized charge ratio retained a monotonic relationship with the point of force application over the entire cable length. Therefore, accurate localization remained possible through data-driven calibration using the smoothing-spline approach. This result indicates that the proposed sensing principle is robust against moderate electrode nonuniformity and does not require perfectly uniform resistance distribution for practical operation. Smoothing-spline calibration compensated for this material nonuniformity and reduced the localization error to 25mm over a 1m cable length.

A rigorous quantitative analysis of the detailed stress distribution within the cable would require a mechanical deformation model that accounts for compression, bending, torsion, vibration, fixture conditions, and cable geometry, potentially using finite-element analysis. However, the primary objective of this study was not to fully solve the mechanical deformation field inside the cable sensor, but rather to demonstrate a practical localization principle capable of identifying the position of strain generation within a distributed piezoelectric capacitive structure.

Despite the simplified treatment of the mechanical response, the experimentally measured charge ratios remained monotonic with loading position and showed good agreement with the theoretical trend predicted by the proposed resistance-based charge-division model. These results indicate that the dominant behavior governing localization is the resistance-dependent division of the generated piezoelectric charge, while the remaining mechanical complexities can be effectively compensated through calibration using the smoothing-spline approach.

For wider-area deployment, the proposed method has the advantage of using a single continuous sensing element and two readout channels. Because the localization principle is based on the normalized charge ratio rather than the absolute charge magnitude, moderate variations in signal amplitude due to environmental factors may have only a limited influence on position estimation. However, calibration stability under repeated bending, long-term use, temperature variation, and different installation conditions should be evaluated in future work.

In particular, mechanical bending may modify the local resistance distribution of the high-resistance electrode and alter the charge-division characteristics. Similarly, thermal expansion, viscoelastic deformation of the polymer structure, and long-term material degradation may influence both the electrode resistance and piezoelectric response. Repeated loading may also affect the mechanical contact condition between the electrode and polymer layer, potentially causing gradual drift in the calibration characteristics over time.

In practical implementations, cable fixation conditions and bending radius are also expected to influence the stress distribution inside the cable sensor and may therefore modify the generated charge distribution. Nevertheless, because the proposed method utilizes a data-driven calibration relationship based on the measured charge ratio, recalibration procedures could compensate for moderate environmental or installation-induced variations. Further improvement in electrode uniformity would also enable the system to approach the ideal linear relation in (3), reducing the number of calibration data required for deployment.

## 5. Conclusions

In this study, a one-dimensional position detection system was established using a flexible cable piezoelectric sensor with a high-resistance central electrode. The sensing principle, based on the charge-division law, was first verified with a discrete five-segment cable configuration, and correct section prediction was achieved for all sections. In continuous localization experiments, the nonuniform resistivity of the Tyranno fiber electrode caused nonlinear output characteristics. By implementing a smoothing-spline-based calibration model and validating it through fourfold cross-validation, we estimated the point of force application with a root mean square error of 25mm over a 1m sensor length. This corresponds to a relative error of 2.5%, demonstrating the feasibility of high-precision, wide-area sensing using a single continuous element.

## Figures and Tables

**Table 1:** Comparison between theoretical and measured ratios, including the predicted sections for each loaded section.

Loaded Section	Theoretical Ratio	Measured Ratio	Predicted Section
Section 1	1.00	1.010	Section 1
Section 2	0.75	0.760	Section 2
Section 3	0.50	0.518	Section 3
Section 4	0.25	0.258	Section 4
Section 5	0.00	0.002	Section 5

## Data Availability

The data presented in this study are available from the corresponding author upon reasonable request.

## Abbreviations

The following abbreviations are used in this manuscript:

CA	Charge amplifier
IoT	Internet of Things
PET	Polyethylene terephthalate
PVDF	Polyvinylidene fluoride
SiC	Silicon carbide
